# Synthesis and evaluation of anthranilamide-based derivatives as FXa inhibitors

**DOI:** 10.18632/oncotarget.16427

**Published:** 2017-03-21

**Authors:** Changjiang Huang, Wenzhi Wang, Yao Li, Shijun Zhang, Fancui Meng, Weiren Xu, Jing Yuan, Ligong Chen

**Affiliations:** ^1^ School of Chemical Engineering and Technology, Tianjin University, Tianjin, China; ^2^ Tianjin Key Laboratory of Molecular Design and Drug Discovery, Tianjin Institute of Pharmaceutical Research, Tianjin, China; ^3^ School of Pharmaceutical Engineering, Shenyang Pharmaceutical University, Shenyang, China; ^4^ Collaborative Innovation Center of Chemical Science and Engineering, Tianjin, China; ^5^ Tianjin Engineering Research Center of Functional Fine Chemicals, Tianjin, China

**Keywords:** thrombosis, thromboembolic diseases, anticoagulants, factor Xa inhibitors, thrombin and docking simulation

## Abstract

Factor Xa (FXa) plays a significant role in the blood coagulation cascade and is a promising target for anticoagulation drugs. Three oral FXa inhibitors have been approved by FDA for treating thrombotic diseases. In this study, 43 novel compounds were synthesized anthranilamide-based FXa inhibitors aiming to ameliorate the toxicity of traditional FXa inhibitors in clinic. The data indicated that the compounds 6a, 6a-b, 6a-e, 6k, 6k-a and 6k-b showed remarkable FXa inhibitory activity and excellent selectivity over thrombin *in vitro*. Selected compounds also exhibited anticoagulant activities *in vitro* consequently and were potent novel anti-coagulators in further.

## INTRODUCTION

**Figure 1:**
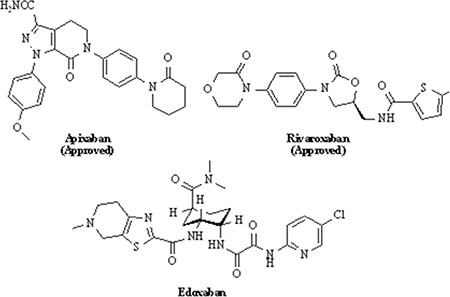
Oral direct FXa inhibitors

The coagulation and anticoagulation process was considered as a dynamic equilibrium process, the breakage of this balance was capable to induce thrombosis [1]. Warfarin was employed as oral anticoagulant for decades [2] however the clinical utility of warfarin was limited by its narrow therapeutic index, dietary restrictions, slow onset of action and the need for regular monitoring [3, 4]. In order to overcome this clinical obstacle of warfarin, researchers focused on developing novel synthetic molecules to inhibit specific enzymes such as protease factor Xa (FXa) [5]. FXa is a junction of the intrinsic and extrinsic pathways, and it was convinced to be a key component in the coagulation factor activation and thrombosis formation [6]. Up to date, three oral direct FXa inhibitors such as Rivaroxaban have been approved to treat the venous thrombosis in clinics and several other candidates were in the variant stages of clinical studies or biological testing (the structures of three approved drugs were in Figure 1) [7]. However, these three medicines still possess specific obstacles in clinical utility, such as risk of bleeding (including intracranial bleeding, gastrointestinal bleeding, epistaxis and other fatal bleeds), no antidote and higher incidence of thromboembolic events after ceasing treatment [8]. Therefore, the development of novel antithrombotic drugs is still attracted many attention for unmet clinical demand.

According to structural analysis, the carboxamide group at both rivaroxaban and betrixaban was considered to play a crucial role for connecting the scaffold, P1 and P4. Betrixaban interacts with residues Gly218 and Gly216 in FXa by two hydrogen bonds [7]. All these structural information supported a hypothesis that the carboxamide group might be crucial component for the docking of FXa inhibitors with human FXa protein. In this study, we synthesized 43 novel compounds in which carboxamide group was involved.

Additionally, the clinical pharmacokinetics investigations of rivaroxaban revealed that the metabolic pathway of rivaroxaban is to hydrolyze the amide bond and then eliminate via renal and biliary/fecal routes rapidly [9]. The metabolic characterizations of rivaroxaban suggested less toxicity by reversing the order of chemical group in betrixaban which was presumed to result in totally different metabolic pathways in humans even. In brief, we designed and evaluated novel synthesized compounds as inhibitors of FXa and their structural information upon computational simulation approaches.

## RESULTS AND DISCUSSION

### Synthesis

**Figure 2:**
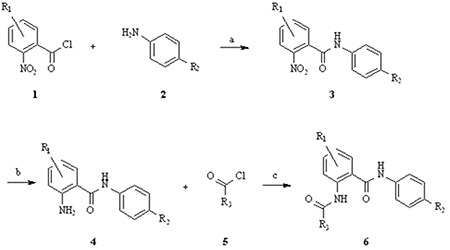
Synthesis of Compound 6 Reagent and conditions: (**A**) THF, K_2_CO_3_, DMAP, reflux, 2 h; (**B**) Zn, NH_4_Cl, H_2_O, THF, MeOH, 40°C, 2 h; (**C**) THF, K_2_CO_3_, DMAP, reflux, 4 h.

The compounds listed in Tables 1 and 2 are prepared as shown in Figure 2. The compound 3 was synthesized from the anilines 2 and o-nitrobenzoyl chloride 1, Then the anilines 4 was prepared by reduction reaction. Compound 6 was accessed by treatment of compound 4 with compound 5. The compound's Activity for inhibit FXa was reported as IC50 and we also reported the Ki of several more potent compounds.

**Table 1:** Representative SAR for the P1 variants

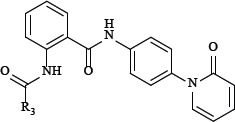		
No.	R_3_	FXa IC_50_ (nM)
**6a**	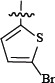	28.7
**6b**	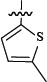	> 1000
**6c**	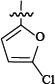	> 1000
**6d**	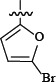	> 1000
**6e**	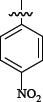	> 1000
**6f**	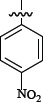	> 1000
		
**6g**	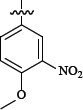	> 1000
**6h**	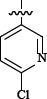	277.5
**6i**	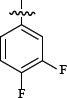	> 1000
**6j**	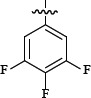	> 1000
**6k**	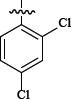	23.8
**6l**	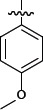	181.5
**6m**	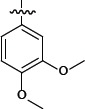	> 1000
**6n**	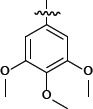	> 1000
**6o**	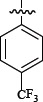	> 1000

**Table 2:** SAR of substituent on benzene ring and different P4 group

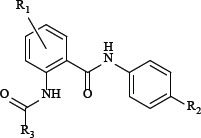				
No.	R_1_	R_2_	R_3_	FXa IC_50_ (nM)
**6a-a**	5-chloro	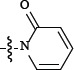	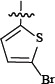	121.0
**6a-b**	5-methyl			18.8
**6a-c**	3-methyl			>1000
**6a-d**	H	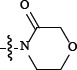		113.4
**6a-e**	5-chloro			125.0
**6a-f**	5-methyl			54.8
**6a-g**	3-methyl			>1000
**6h-a**	5-chloro	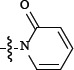	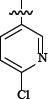	427.4
**6h-b**	5-methyl			212.3
**6h-c**	3-methyl			>1000
**6h-d**	H	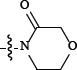		>1000
**6h-e**	5-chloro			780.8
**6h-f**	5-methyl			453.0
**6h-g**	3-methyl			>1000
**No**.	**R_1_**	**R_2_**	**R_3_**	**FXa IC_50_ (nM)**
**6k-a**	5-chloro	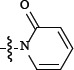	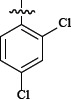	82.0
**6k-b**	5-methyl			35.5
**6k-c**	3-methyl			>1000
**6k-d**	H	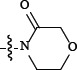		127.5
**6k-e**	5-chloro			259.3
**6k-f**	5-methyl			282.4
**6k-g**	3-methyl			>1000
**6l-a**	5-chloro	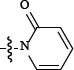	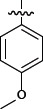	>1000
**6l-b**	5-methyl			452.1
**6l-c**	3-methyl			>1000
**6l-d**	H	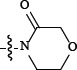		459.3
**6l-e**	5-chloro			484.6
**6l-f**	5-methyl			433.1
**6l-g**	3-methyl			>1000

### *In Vitro* inhibition activity studies on FXa

We extensively investigated different P1 group. Important SAR findings are summarized in Table 1. Compounds 6a-6o that contained same scaffold and P4 group were used for choosing potent P1 group. Compounds 6a, 6h, 6k and 6l exhibited a promising inhibitory activity against FXa with IC_50_ values of 28.7, 277.5, 23.8 and 181.5 nM. The P1 group of 6a, 6h, 6k and 6l were chosen for structural modification of next stage and provided an opportunity to improve potency.

Then, the SAR of substituent on benzene ring and different P4 group were examined (Table 2). Unfortunately, the 2-chloropyridine analogs (6h-a – 6h-g) and 4-methoxybenzene analogs (6l-a – 6l-g) still did not exhibited good activity. However, 5-bromothiophene analogs and 2, 4-dichlorobenzene analogs both showed excellent activity. In particular compounds 6a-b and 6k-b showed some validity with the IC_50_ value of 18.8 and 35.5 nM. From these results we can find that 3-methyl-substituted scaffold (6a-c, 6a-g, 6k-c and 6k-g) was not suitable, the related compounds displayed poor IC_50_ values at a micromole level no matter what kind of R_2_ and R_3_. Compounds with 5-electron donating group-substituted scaffold exhibited almost 10 fold better than 5-electron withdrawing group substituted scaffold. The IC_50_ value of compounds with non-substituted scaffold was almost at the same level with electron donating group scaffold.

### Thrombin selectivity comparison and assay of prothrombin time (PT)

Table 3 shows the thrombin selectivity comparison and the extension of the prothrombin time (PT) of 6a, 6a-b, 6a-e, 6k, 6k-a and 6k-b. The IC50 of rivaroxaban against thrombin is 6.9 μM [10], our compounds were also highly selective, IC_50_ were all far higher than 10 μM. The assay of PT also showed that our compound possessed improvement on anticoagulant selectivity.

**Table 3:** Anticoagulant activity of 6a, 6a-b, 6a-e, 6k, 6k-a and 6k-b

No.	FXa *Ki* (nM)	2 × PT (μM) (Human)
**6a**	20.5	8.4
**6a-b**	13.4	4.2
**6a-e**	39.1	3.8
**6k**	17.0	16.4
**6k-a**	58.6	16.9
**6k-b**	25.4	11.3
**rivaroxaban**	0.7	0.2

### Docking simulation studies

**Figure 3:**
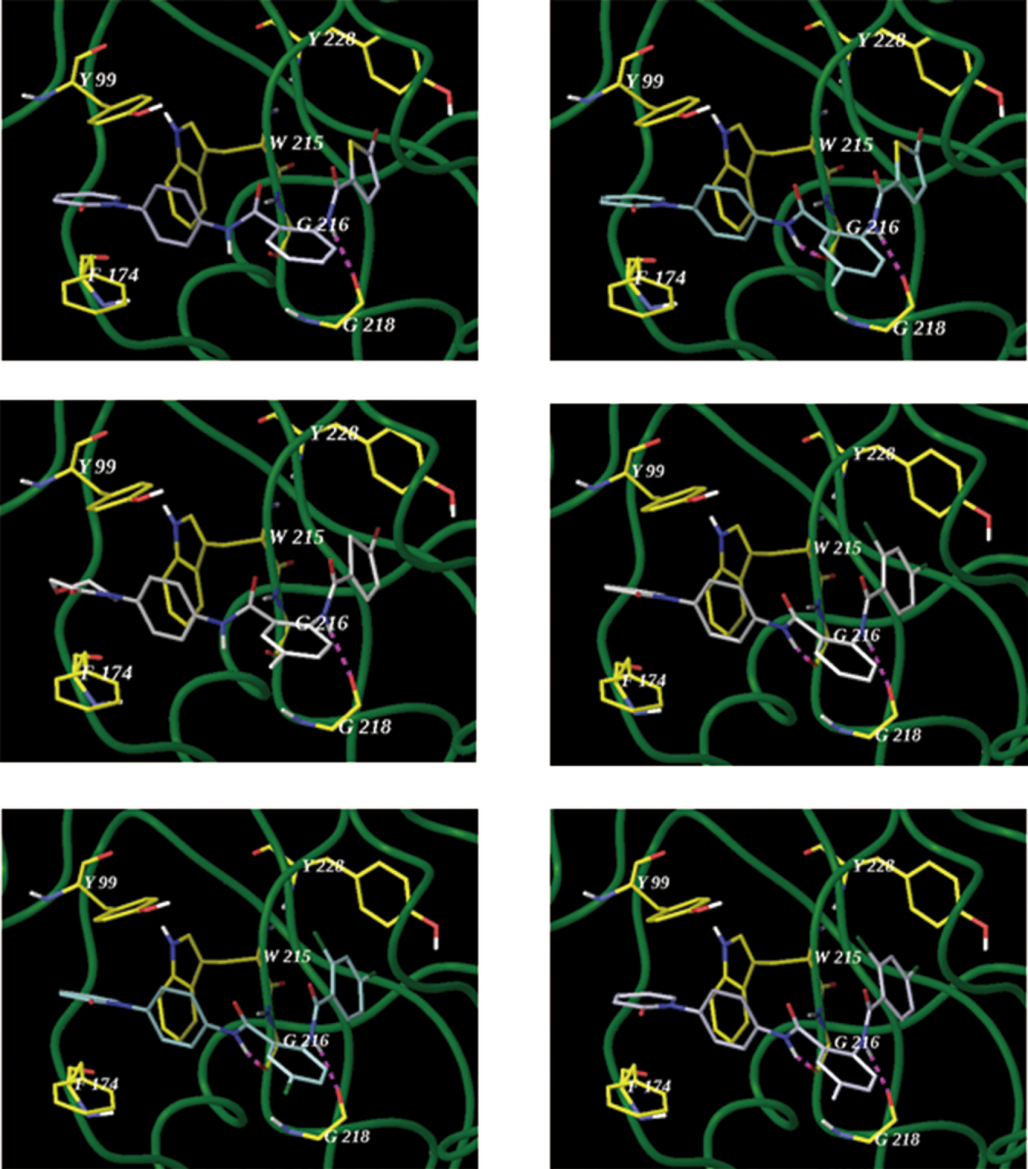
The interactions of compounds 6a (1st panel, PDB code 2xbv), 6a-b (2nd panel), 6a-e (3rd panel), 6k (4th panel), 6k-a (5th panel), 6k-b (6th panel) with the active site of FXa

In order to analyze the detailed interactions between compounds 6a, 6a-b, 6a-e, 6k, 6k-a and 6k-b to FXa, the computational docking simulation studies were performed. As showed in Figure 3, There are residue Gly216 and Gly218 in S1 pocket of FXa, and some hydrogen bonds were taked shape betweed conpound and these amino-acid residue. the unit of pyridone or morpholino are access the S4 pocket of FXa, which was composed of Tyr99, Phe174 and Trp215. The p-p conjugation was formed between the chloro- or bromo- substituent and S1 pocket through amino-acid residue Tyr228. The mode of action was similar with rivaroxaban [7].

## MATERIALS AND METHODS

Reagents and solvents were obtained from commercial suppliers and used as received without further purification. All reactions were monitored by thin layer chromatography. ^1^H NMR spectra (400 MHz) were recorded for DMSO-*d*6 solutions on an AV400 NMR (Bruker, Billerica, MA, USA), MS were measured on a Finnigan LCQ Mass (Thermo Fisher Scientific, Cambridge, MA, USA), HRMS were measured on a miorOTOF-QII instrument (Bruker Daltonics, Billerica, MA, USA) and melting points (uncorrected) were determined on a YRT-3 Melting Point Tester (Precision Instrument of Tianjin University, Tianjin, China).

### Synthesis of 3 (3a-3h)

To a stirred solution of compound 2 (4.8 mmol) (2a-2b), K_2_CO_3_ (0.80 g, 5.8 mmol) and DMAP (0.05 g, 0.4 mmol) in THF (20 mL), solution of compound 1 (6.24 mmol) (1a-1d) in THF (5 mL) was added at room temperature and the mixture was refluxed for 2 h. The reaction mixture was cooled down to room temperature and concentrated under reduced pressure. Then water (100 mL) was added to the mixture and stirred for 10 min at room temperature. The resulting precipitate was collected by filtration. The reaction was monitored by TLC with EA.

### Synthesis of 4 (4a-4h)

To a 250 mL round bottom flask, compound 3 (3.9 mmol) (3a-3h), zinc powder (2.05 g, 31.2 mmol), NH_4_Cl (2.11 g, 39 mmol), methanol (30 mL), THF (30 mL) and water (15 mL) were added. The mixture stirred at 40°C for 2 h. The reaction mixture was filtered, washed with DMF and the filtrate was concentrated under reduced pressure. Then water (200 mL) was added to the mixture and stirred for 0.5 h. The residue was filtered and washed with water to yield the title compound 4 (4a-4h). The reaction was monitored by TLC with EA.

### Synthesis of 6 (6a-6o, 6a-a–6a-g, 6h-a–6h-g, 6h-a–6h-g, 6l-a–6l-g)

To a stirred solution of compound 4 (0.98 mmol) (4a-4h), K_2_CO_3_ (0.16 g, 1.18 mmol) and DMAP (0.01 g, 0.08 mmol) in THF (10 mL), solution of compound 5 (1.27 mmol) (5a-5h) in THF (5 mL) was added at room temperature and the mixture was refluxed for 4 h. The reaction mixture was cool down to room temperature and water (30 mL) was added. The resulting precipitate was collected by filtration. The authentic sample was prepared by recrystallization from DMF/MeOH. The reaction was monitored by TLC with EA.

### Spectral date

### 2-Nitro-N-(4-(2-oxopyridin-1(2H)-yl)phenyl)benzami-de (3a)

White solid product (1.46 g, 90%). MS: [M + H]^+^ 336.22. ^1^H NMR: δ ppm 6.30 (t, *J* = 6.4 Hz, 1H), 6.47 (d, *J* = 9.2 Hz, 1H), 7.39 (d, *J* = 8.8 Hz, 2H), 7.49 (t, *J* = 4.4 Hz, 1H), 7.63 (d, *J* = 6.8 Hz, 1H), 7.76–7.80 (m, 4H), 7.89 (t, *J* = 7.6 Hz, 1H), 8.17 (d, *J* = 8.0 Hz, 1H), 10.84 (s, NH).

### 5-Chloro-2-nitro-N-(4-(2-oxopyridin-1(*2H*)-yl)phenyl)-benzamide (3b)

White solid product (1.64 g, 92%). MS: [M + H]^+^ 370.05. ^1^H NMR: δ ppm 6.31 (t, *J* = 6.8 Hz, 1H), 6.47 (d, *J* = 9.2 Hz, 1H), 7.40 (d, *J* = 8.4 Hz, 2H), 7.50 (t, *J* = 2.4 Hz, 1H), 7.63 (d, *J* = 8.8 Hz, 1H), 7.76 (d, *J* = 6.8 Hz, 2H), 7.86 (d, *J* = 6.4 Hz, 1H), 8.00 (s, 1H), 8.20 (d, *J* = 8.8 Hz, 1H), 10.88 (s, NH).

5-Methyl-2-nitro-N-(4-(2-oxopyridin-1(2H)-yl)phenyl) benzamide (3c): White solid product (1.50 g, 89%). MS: [M + H]^+^ 350.42. ^1^H NMR: δ ppm 2.43 (s, 3H), 6.31 (t, *J* = 6.4 Hz, 1H), 6.47 (d, *J* = 9.2 Hz, 1H), 7.38 (d, *J* = 8.8 Hz, 2H), 7.50 (t, *J* = 4.8 Hz, 1H), 7.55–7.64 (m, 3H), 7.76 (d, *J* = 9.2 Hz, 2H), 8.08 (d, *J* = 8.4 Hz, 1H), 10.78 (s, NH).

### 3-Methyl-2-nitro-N-(4-(2-oxopyridin-1(*2H*)-yl)phenyl) benzamide (3d)

White solid product (1.54 g, 91%). MS: [M + H]^+^ 350.1. ^1^H NMR: δ ppm 2.36 (s, CH_3_), 6.30 (t, *J* = 6.4 Hz, 1H), 6.46 (d, *J* = 9.2 Hz, 1H), 7.38–7.40 (m, 2H), 7.49 (t, *J* = 8.8 Hz,1H), 7.62 (d, *J* = 8.8 Hz, 1H), 7.64–7.67 (m, 2H), 7.69–7.73 (m, 1H),7.77 (d, *J* = 8.8 Hz, 2H), 10.87 (s, NH).

### 2-Nitro-N-(4-(3-oxomorpholino)phenyl)benzamide (3e)

White solid product (1.54 g, 93 %). MS: [M + H]^+^ 342.09. ^1^H-NMR: δ ppm 3.71 (t, *J* = 4.8 Hz, CH_2_), 3.97 (t, *J* = 4.8 Hz, CH_2_), 4.19 (s, 2H), 7.37 (d, *J* = 8.8 Hz, 2H), 7.67 (d, *J* = 8.8 Hz, 2H), 7.74–7.78 (m, 2H), 7.87 (t, *J* = 7.6 Hz, 1H), 8.15 (d, *J* = 8.0 Hz, 1H), 10.72 (s, NH).

### 5-Chloro-2-nitro-N-(4-(3-oxomorpholino)phenyl)benz-amide (3f)

White solid product (1.67 g, 94%). MS: [M + H]^+^ 376.05. ^1^H NMR: δ ppm 3.72 (t, *J* = 10.0 Hz, 2H), 3.97 (t, *J* = 10.0 Hz, 2H), 4.19 (s, 2H), 7.38 (d, *J* = 8.4 Hz, 2H), 7.66 (d, *J* = 8.8 Hz, 2H), 7.84 (d, *J* = 8.8 Hz, 2H), 7.94 (s, 1H), 8.18 (d, *J* = 8.8 Hz, 2H), 10.77 (s, NH).

### 5-Methyl-2-nitro-N-(4-(3-oxomorpholino)phenyl)benz-amide (3g)

White solid product (1.55 g, 90%). MS: [M + H]^+^ 355.99. ^1^H NMR: δ ppm 2.47 (s, CH_3_), 3.71 (t, *J* = 4.8 Hz, CH_2_), 3.97 (t, *J* = 4.8 Hz, CH_2_), 4.19 (s, CH_2_), 7.37 (d, *J* = 8.8 Hz, 2H), 7.55 (d, *J* = 8.8 Hz, 2H), 7.57 (s, 1H), 7.67 (d, *J* = 8.8 Hz, 2H), 8.07 (d, *J* = 8.4 Hz, 1H), 10.66 (s, NH).

### 3-Methyl-2-nitro-N-(4-(3-oxomorpholino)phenyl)benz-amide (3h)

White solid product (1.53 g, 89%). MS: [M + H]^+^ 356.19. ^1^H NMR: δ ppm 2.36 (s, CH_3_), 3.72 (t, *J* = 4.8 Hz, CH_2_), 3.98 (t, *J* = 4.8 Hz, CH_2_), 4.20 (s, CH_2_), 7.38 (d, *J* = 8.8 Hz, 2H), 7.65–7.24 (m, 5H), 10.76 (s, NH).

### 2-Amino-N-(4-(2-oxopyridin-1(*2H*)-yl)phenyl)benzam-ide (4a)

White solid product (1.13 g, 89%). MS: [M + H]^+^ 306.10. ^1^H NMR: δ ppm 6.30 (t, *J* = 6.4 Hz, 1H), 6.34 (s, NH_2_), 6.46 (d, *J* = 8.8 Hz, 1H), 6.60 (t, *J* = 7.2 Hz, 1H), 6.76 (d, *J* = 8.4 Hz, 1H), 7.21 (t, *J* = 7.2 Hz, 1H), 7.33–7.36 (m, 2H), 7.49 (t, *J* = 8.8 Hz, 1H), 7.61–7.65 (m, 2H), 7.83 (d, *J* = 8.8 Hz, 2H), 10.14 (s, NH).

### 2-Amino-5-chloro-N-(4-(2-oxopyridin-1(*2H*)-yl)phenyl) benzamide (4b)

Yellow solid product (1.29 g, 91%). MS: [M + H]^+^ 340.10. ^1^H NMR: δ ppm 6.30 (t, *J* = 5.2 Hz, 1H), 6.47 (d, *J* = 10.4 Hz, 1H), 6.48 (s, NH_2_), 6.78 (d, *J* = 9.2 Hz, 1H), 7.24 (d, *J* = 6.8 Hz, 1H), 7.35 (d, *J* = 8.8 Hz, 2H), 7.49 (t, *J* = 7.2 Hz, 1H), 7.63 (d, *J* = 8.4 Hz, 1H), 7.70 (s, 1H), 7.80 (d, *J* = 8.8 Hz, 2H), 10.24 (s, NH).

### 2-Amino-5-methyl-N-(4-(2-oxopyridin-1(*2H*)-yl)phenyl) benzamide (4c)

Yellow solid product (1.23 g, 92%). MS: [M + H]^+^ 320.04. ^1^H NMR: δ ppm 2.22 (s, CH_3_), 6.11 (s, NH_2_), 6.30 (t, *J* = 6.4 Hz, 1H), 6.46 (d, *J* = 8.8 Hz, 1H), 6.68 (d, *J* = 8.4 Hz, 1H), 7.04 (d, *J* = 8.0 Hz, 1H), 7.34 (d, *J* = 8.8 Hz, 2H), 7.44 (s, 1H), 7.49 (t, *J* = 8.8 Hz, 1H), 7.63 (d, *J* = 8.8 Hz, 1H), 7.81 (d, *J* = 8.8 Hz, 2H), 10.11 (s, NH).

### 2-Amino-3-methyl-N-(4-(2-oxopyridin-1(*2H*)-yl)phenyl) benzamide (4d)

White solid product (1.20 g, 90%). MS: [M + H]^+^ 320.04. ^1^H NMR: δ ppm 2.12 (s, CH_3_), 6.13 (s, NH_2_), 6.30 (t, *J* = 6.8 Hz, 1H), 6.46 (d, *J* = 9.2 Hz, 1H), 6.58 (t, *J* = 7.2 Hz, 1H), 7.15 (d, *J* = 7.2 Hz, 1H), 7.34 (d, *J* = 8.8 Hz, 2H), 7.47–7.54 (m, 2H), 7.62 (d, *J* = 8.4 Hz, 1H), 7.82 (d, *J* = 8.8 Hz, 2H), 10.17 (s, NH).

### 2-Amino-N-(4-(3-oxomorpholino)phenyl)benzamide (4e)

White solid product (1.21 g, 93%). MS: [M + H]^+^ 312.04. ^1^H NMR: δ ppm 3.71 (t, *J* = 4.8 Hz, CH_2_), 3.96 (t, *J* = 4.8 Hz, CH_2_), 4.18 (s, CH_2_), 6.31 (s, NH_2_), 6.58 (t, *J* = 7.2 Hz, 1H), 6.74 (d, *J* = 8.0 Hz, 1H), 7.13 (t, *J* = 8.4 Hz, 1H), 7.33 (d, *J* = 7.2 Hz, 2H), 7.61 (d, *J* = 8.8 Hz, 1H), 7.72 (d, *J* = 8.8 Hz, 2H), 10.03 (s, NH).

### 2-Amino-5-chloro-N-(4-(3-oxomorpholino)phenyl)ben-zamide (4f)

White solid product (1.28 g, 89%). MS: [M + H]^+^ 345.97. ^1^H NMR: δ ppm 3.71 (t, *J* = 4.8 Hz, CH_2_), 3.96 (t, *J* = 4.8 Hz, CH_2_), 4.19 (s, CH_2_), 6.46 (s, NH_2_), 6.78 (d, *J* = 8.8 Hz, 1H), 7.23 (d, *J* = 8.8 Hz, 2H), 7.34 (d, *J* = 8.8 Hz, 2H), 7.68 (d, *J* = 7.6 Hz, 2H), 7.72 (s, 1H), 10.14 (s, NH).

### 2-Amino-5-methyl-N-(4-(3-oxomorpholino)phenyl)be-nzamide (4g)

Yellow solid product (1.22 g, 90%). MS: [M + H]^+^ 326.07. ^1^H NMR: δ ppm 2.21 (s, CH_3_), 3.71 (t, *J* = 4.8 Hz, CH_2_), 3.96 (t, *J* = 4.8 Hz, CH_2_), 4.19 (s, CH_2_), 6.09 (s, NH_2_), 6.67 (d, *J* = 8.4 Hz, 1H), 7.03 (d, *J* = 8.0 Hz, 1H), 7.33 (d, *J* = 8.8 Hz, 2H), 7.42 (s, 1H), 7.72 (d, *J* = 8.8 Hz, 2H), 10.01 (s, NH).

### 2-Amino-3-methyl-N-(4-(3-oxomorpholino)phenyl)be-nzamide (4h)

White solid product (1.23 g, 91%). MS: [M + H]^+^ 326.03. ^1^H NMR: δ ppm 2.11 (s, CH_3_), 3.71 (t, *J* = 4.8 Hz, CH_2_), 3.96 (t, *J* = 4.8 Hz, CH_2_), 4.19 (s, CH_2_), 6.11 (s, NH_2_), 6.56 (t, *J* = 7.6 Hz, 1H), 7.13 (d, *J* = 6.8 Hz, 1H), 7.33 (d, *J* = 8.8 Hz, 2H), 7.51 (d, *J* = 7.6 Hz, 1H), 7.72 (d, *J* = 8.8 Hz, 2H), 10.06 (s, NH).

### 5-bromo-N-(2-((4-(2-oxopyridin-1(2H)-yl)phenyl)carb-amoyl)phenyl)thiophene-2-carboxamide (6a)

White solid product (0.26 g, 56%), m.p. > 250°C. ^1^H NMR (400 MHz, DMSO): δ ppm 6.31 (t, *J* = 6.8 Hz, 1H), 6.47 (d, *J* = 8.8 Hz, 1H), 7.31–7.40 (m, 4H), 7.47–7.52 (m, 1H), 7.58–7.64 (m, 3H), 7.81 (d, *J* = 8.4 Hz, 2H), 7.92 (d, *J* = 7.6 Hz, 1H), 8.23 (d, *J* = 8.0 Hz 1H), 10.67 (s, NH), 11.53 (s, NH). HRMS (ESI) calcd. for C_23_H_16_BrN_3_O_3_S: [M + Na]^+^ m/z: 515.9993, found: 515.9998.

### 5-methyl-N-(2-((4-(2-oxopyridin-1(*2H*)-yl)phenyl)carb-amoyl)phenyl)thiophene-2-carboxamide (6b)

Yellow solid product (0.25 g, 60%), m.p. > 250°C. ^1^H NMR (400 MHz, DMSO): δ ppm 2.49 (s, CH_3_), 6.30 (t, *J* = 6.8 Hz, 1H), 6.47 (d, *J* = 9.2 Hz, 1H), 6.93 (d, *J* = 3.2 Hz, 1H), 7.27 (t, *J* = 6.8 Hz, 1H), 7.39 (d, *J* = 8.8 Hz, 2H), 7.47–7.64 (m, 4H), 7.82 (d, *J* = 8.8 Hz, 2H), 7.93 (d, *J* = 8.0 Hz, 1H), 8.36 (d, *J* = 8.0 Hz, 1H), 10.67 (s, NH), 11.53 (s, NH). HRMS (ESI) calcd. for C_24_H_19_N_3_O_3_S: [M + Na]^+^ m/z: 452.1045, found: 452.1032.

### 5-chloro-N-(2-((4-(2-oxopyridin-1(*2H*)-yl)phenyl)carb-amoyl)phenyl)furan-2-carboxamide (6c)

White solid product (0.24 g, 59%), m.p. > 250°C. ^1^H NMR (400 MHz, DMSO): δ ppm 6.31 (t, *J* = 6.4 Hz, 1H), 6.47 (d, *J* = 9.2 Hz, 1H), 6.76 (d, *J* = 8.4 Hz, 1H), 7.33–7.48 (m, 4H), 7.50 (t, *J* = 7.2 Hz, 1H), 7.62–7.67 (m, 2H), 7.83 (d, *J* = 7.6 Hz, 2H), 7.92 (d, *J* = 7.6 Hz, 1H), 8.34 (d, *J* = 8.0 Hz, 1H), 10.71 (s, NH), 11.47 (s, NH). HRMS (ESI) calcd. for C_23_H_16_ClN_3_O_4_: [M + Na]^+^ m/z: 456.0727, found: 456.0728.

### 5-bromo-N-(2-((4-(2-oxopyridin-1(*2H*)-yl)phenyl)carb-amoyl)phenyl)furan-2-carboxamide (6d)

White solid product (0.30 g, 65%), m.p. > 250°C. ^1^H NMR (400 MHz, DMSO): δ ppm 6.31 (t, *J* = 6.4 Hz, 1H), 6.47 (d, *J* = 9.6 Hz, 1H), 6.85 (d, *J* = 3.2 Hz, 1H), 7.29–7.33 (m, 2H), 7.41 (d, *J* = 8.4 Hz, 2H), 7.48–7.53 (m, 1H), 7.61 (d, *J* = 7.6 Hz, 1H), 7.65 (d, *J* = 6.4 Hz, 1H), 7.83 (d, *J* = 8.4 Hz, 2H), 7.92 (d, *J* = 7.6 Hz, 1H), 8.34 (d, *J* = 8.0 Hz, 1H), 10.71 (s, NH), 11.46 (s, NH). HRMS (ESI) calcd. for C_23_H_16_BrN_3_O_4_: [M + Na]^+^ m/z: 500.0222, found: 500.0231.

### 2-(4-nitrobenzamido)-N-(4-(2-oxopyridin-1 (*2H*)-yl)ph-enyl)benzamide (6e)

Yellow solid product (0.30 g, 70%), m.p. > 250°C. ^1^H NMR (400 MHz, DMSO): δ ppm 6.30 (t, *J* = 6.4 Hz, 1H), 6.46 (d, *J* = 9.2 Hz, 1H), 7.37 (d, *J* = 8.8 Hz, 3H), 7.49 (t, *J* = 8.8 Hz, 1H), 7.60–7.75 (m, 2H), 7.80 (d, *J* = 8.8 Hz, 2H), 7.92 (d, *J* = 7.6 Hz, 1H), 8.14 (d, *J* = 8.4 Hz, 2H), 8.29 (d, *J* = 8.4 Hz, 1H), 8.39 (d, *J* = 8.8 Hz, 2H), 10.67 (s, NH), 11.62 (s, NH). HRMS (ESI) calcd. for C_26_H_18_N_4_O_3_: [M + Na]^+^ m/z: 477.1175, found: 477.1165.

### 2-(4-cyanobenzamido)-N-(4-(2-oxopyridin-1(*2H*)-yl) phenyl)benzamide (6f)

Black solid product (0.24 g, 57%), m.p. > 250°C. ^1^H NMR (400 MHz, DMSO): δ ppm 6.30 (t, *J* = 6.4 Hz, 1H), 6.47 (d, *J* = 9.2 Hz, 1H), 7.39–7.32 (m, 3H), 7.49 (t, *J* = 8.8 Hz, 1H), 7.62 (d, *J* = 7.2 Hz, 2H), 7.80 (d, *J* = 8.8 Hz, 2H), 7.92 (d, *J* = 8.0 Hz, 1H), 8.06 (d, *J* = 8.4 Hz, 4H), 8.32 (d, *J* = 8.0 Hz, 1H), 10.69 (s, NH), 11.60 (s, NH). HRMS (ESI) calcd. for C_26_H_18_N_4_O_3_: [M + Na]^+^ m/z: 457.1277, found: 457.1282.

### 4-methoxy-3-nitro-N-(2-((4-(2-oxopyridin-1(*2H*)-yl)ph-enyl)carbamoyl)phenyl)benzamide (6g)

White solid product (0.30 g, 65%), m.p. > 250°C. ^1^H NMR (400 MHz, DMSO): δ ppm 4.0 (s, CH_3_), 6.30 (t, *J* = 6.8 Hz, 1H), 6.47 (d, *J* = 8.8 Hz, 1H), 7.31–7.39 (m, 3H), 7.49 (t, *J* = 8.8 Hz, 1H), 7.57 (d, *J* = 9.2 Hz,1H), 7.60–7.64 (m, 2H), 7.81 (d, *J* = 8.8 Hz, 2H), 7.90 (d, *J* = 8.8 Hz, 1H), 8.17 (d, *J* = 8.8 Hz, 1H), 8.24 (d, *J* = 8.24 Hz, 1H), 8.42 (s, 1H), 10.66 (s, NH), 11.46 (s, NH). HRMS (ESI) calcd. for C_26_H_20_N_4_O_6_: [M + Na]^+^ m/z: 507.1281, found: 507.1281.

### 6-chloro-N-(2-((4-(2-oxopyridin-1(*2H*)-yl)phenyl)carb-amoyl)phenyl)nicotinamide (6h)

Yellow solid product (0.26 g, 62%), m.p. > 250°C. ^1^H NMR (400 MHz, DMSO): δ ppm 6.30 (t, *J* = 6.4 Hz, 1H), 6.46 (d, *J* = 9.2 Hz, 1H),7.33–7.38 (m, 3H), 7.49 (t, *J* = 8.8 Hz, 1H), 7.60–7.64 (m, 2H), 7.73 (d, *J* = 8.4 Hz, 1H), 7.81 (d, *J* = 8.4 Hz, 2H), 7.90 (d, *J* = 7.6 Hz, 1H), 8.22 (d, *J* = 8.0 Hz, 1H), 8.28 (d, *J* = 8.4 Hz, 1H), 8.90 (s, 1H), 10.67 (s, NH), 11.47 (s, NH). HRMS (ESI) calcd. for C_24_H_17_ClN_4_O_3_: [M + Na]^+^ m/z: 467.0887, found: 467.0882.

### 3,4-difluoro-N-(2-((4-(2-oxopyridin-1(*2H*)-yl)phenyl) carbamoyl)phenyl)benzamide (6i)

White solid product (0.24 g, 60%), m.p. > 250°C. ^1^H NMR (400 MHz, DMSO): δ ppm 6.30 (t, *J* = 6.4 Hz, 1H), 6.46 (d, *J* = 9.2 Hz, 1H), 7.33 (d, *J* = 7.6 Hz, 1H), 7.38 (d, *J* = 8.4 Hz, 2H), 7.49 (t, *J* = 8.8 Hz, 1H), 7.60–7.68 (m, 3H), 7.80 (d, *J* = 8.4 Hz, 3H), 7.90–7.94 (m, 2H), 8.27 (d, *J* = 8.4 Hz, 1H), 10.67 (s, NH), 11.47 (s, NH). HRMS (ESI) calcd. for C_25_H_17_F_2_N_3_O_3_: [M + Na]^+^ m/z: 468.1136, found: 468.1133.

### 3,4,5-trifluoro-N-(2-((4-(2-oxopyridin-1(*2H*)-yl)phenyl)arbamoyl)phenyl)benzamide (6j)

White solid product (0.25 g, 59%), m.p. > 250°C. ^1^H NMR (400 MHz, DMSO): δ ppm 6.38 (t, *J* = 6.4 Hz, 1H), 6.46 (d, *J* = 8.8 Hz, 1H), 7.37 (d, *J* = 8.8 Hz, 3H), 7.49 (t, *J* = 8.8 Hz, 1H), 7.62 (d, *J* = 8.4 Hz, 2H), 7.79–7.90 (m, 4H), 7.89 (d, *J* = 8.0 Hz, 1H), 8.13 (d, *J* = 8.0 Hz 1H), 10.65 (s, NH), 11.31 (s, NH). HRMS (ESI) calcd. for C_25_H_16_F_3_N_3_O_3_: [M + Na]^+^ m/z: 486.1041, found: 486.1031.

### 2,4-dichloro-N-(2-((4-(2-oxopyridin-1(*2H*)-yl)phenyl)arbamoyl)phenyl)benzamide (6k)

White solid product (0.26 g, 61%), m.p. > 250°C. ^1^H NMR (400 MHz, DMSO): δ ppm 6.30 (t, *J* = 6.4 Hz, 1H), 6.46 (d, *J* = 9.2 Hz, 1H), 7.35 (d, *J* = 7.6 Hz, 3H), 8.49 (t, *J* = 8.0 Hz, 1H), 7.56–7.67 (m, 4H), 7.75–7.84 (m, 4H), 8.14 (d, *J* = 6.8 Hz, 1H), 10.64 (s, NH), 10.99 (s, NH). HRMS (ESI) calcd. for C_25_H_17_Cl_2_N_3_O_3_: [M + Na]^+^ m/z: 500.0545, found: 500.0536.

### 2-(4-methoxybenzamido)-N-(4-(2-oxopyridin-1(*2H*)-yl)phenyl)benzamide (6l)

White solid product (0.22 g, 56%), m.p. > 250°C. ^1^H NMR (400 MHz, DMSO): δ ppm 3.82 (s, CH_3_), 6.30 (t, *J* = 6.8 Hz, 1H), 6.47 (d, *J* = 9.2 Hz, 1H), 7.10 (d, *J* = 8.8 Hz, 2H), 7.28 (t, *J* = 7.2 Hz, 1H), 7.39 (d, *J* = 8.8 Hz, 2H), 7.50 (t, *J* = 8.8 Hz, 1H), 7.59–7.64 (m, 2H), 7.82 (d, *J* = 8.8 Hz, 2H), 7.89 (d, *J* = 8.8 Hz, 2H), 7.94 (d, *J* = 7.6, 1H), 8.49 (d, *J* = 8.0 Hz, 1H), 10.69 (s, NH), 11.60 (s, NH). HRMS (ESI) calcd. for C_26_H_21_N_3_O_4_: [M + Na]^+^ m/z: 462.1430, found: 462.1430.

### 3,4-dimethoxy-N-(2-((4-(2-oxopyridin-1(2H)-yl)phenyl)arbamoyl)phenyl)benzamide (6m)

White solid product (0.24 g, 58%), m.p. > 250°C. ^1^H NMR (400 MHz, DMSO): δ ppm 3.83 (s, CH_3_), 6.30 (t, *J* = 6.4 Hz, 1H), 6.47 (d, *J* = 9.2 Hz, 1H), 7.14 (d, *J* = 8.4 Hz, 1H), 7.28 (t, *J* = 7.6 Hz, 1H), 7.39 (t, *J* = 8.4 Hz, 2H), 7.48–7.52 (m, 3H), 7.60–7.63 (m, 2H), 7.84 (d, *J* = 8.8 Hz, 2H), 7.92 (d, *J* = 7.6 Hz, 1H), 8.47 (d, *J* = 8.4 Hz, 1H), 10.69 (s, NH), 11.59 (s, NH). HRMS (ESI) calcd. for C_27_H_23_N_3_O_5_: [M + Na]^+^ m/z: 492.1535, found: 492.1531.

### 3,4,5-trimethoxy-N-(2-((4-(2-oxopyridin-1(*2H*)-yl)phenyl)carbamoyl)phenyl)benzamide (6n)

White solid product (0.28 g, 63%), m.p. > 250°C. ^1^H NMR (400 MHz, DMSO): δ ppm 3.84 (s, CH_3_), 3.72 (s, CH_3_), 6.30 (t, *J* = 6.4 Hz, 1H), 6.46 (d, *J* = 9.2 Hz, 1H), 7.23 (s, 2H), 7.31 (t, *J* = 8.0 Hz, 1H), 7.37 (d, *J* = 8.8 Hz, 2H), 7.49 (t, *J* = 7.2 Hz, 1H), 7.60–7.61 (m, 2H), 7.86 (d, *J* = 8.4 Hz, 2H), 7.91 (d, *J* = 7.6 Hz, 1H), 8.31 (d, *J* = 8.4 Hz, 1H), 10.65 (s, NH), 11.42 (s, NH). HRMS (ESI) calcd. for C_28_H_25_N_3_O_6_: [M + Na]^+^ m/z: 522.1641, found: 522.1633.

### N-(4-(2-oxopyridin-1(2H)-yl)phenyl)-2-(4-(trifluoro-methyl)benzamido)benzamide (6o)

White solid product (0.27 g, 62%), m.p. > 250°C. ^1^H NMR (400 MHz, DMSO): δ ppm 6.30 (t, *J* = 6.8 Hz, 1H), 6.46 (d, *J* = 9.2 Hz, 1H), 7.33–7.39 (m, 3H), 7.49 (t, *J* = 8.8 Hz, 1H), 7.61–7.66 (m, 2H), 7.80 (d, *J* = 8.8 Hz, 2H), 7.92–7.96 (m, 3H), 8.10 (d, *J* = 8.4 Hz, 2H), 8.34 (d, *J* = 8.4 Hz, 1H), 10.69 (s, NH), 11.62 (s, NH). HRMS (ESI) calcd. for C_26_H_18_F_3_N_3_O_3_: [M + Na]^+^ m/z: 500.1198, found: 500.1191.

### 5-bromo-N-(4-chloro-2-((4-(2-oxopyridin-1(*2H*)-yl)ph-enyl)carbamoyl)phenyl)thiophene-2-carboxamide (6a-a)

White solid product (0.30 g, 59%), m.p. > 250°C. ^1^H NMR (400 MHz, DMSO): δ ppm 6.31 (t, *J* = 6.4 Hz, 1H), 6.47 (d, *J* = 9.2 Hz, 1H), 7.39 (d, *J* = 8.8 Hz, 3H), 7.50 (t, *J* = 8.8 Hz, 1H), 7.59–7.68 (m, 2H), 7.80 (d, *J* = 8.4 Hz, 2H), 7.96 (s, 1H), 8.20 (d, *J* = 8.8 Hz, 1H), 10.73 (s, NH), 11.40 (s, NH). HRMS (ESI) calcd. for C_23_H_15_BrClN_3_O_3_S: [M + Na]^+^ m/z: 549.9604, found: 549.9588.

### 5-bromo-N-(4-methyl-2-((4-(2-oxopyridin-1(2H)-yl)ph-enyl)carbamoyl)phenyl)thiophene-2-carboxamide (6a-b)

White solid product (0.26 g, 54%), m.p. > 250°C. ^1^H NMR (400 MHz, DMSO): δ ppm 2.39 (s, CH_3_), 6.31 (t, *J* = 6.4 Hz, 1H), 6.47 (d, *J* = 9.2 Hz, 1H), 7.36–7.43 (m, 4H), 7.50 (t, *J* = 8.8 Hz, 1H), 7.57 (d, *J* = 4.0 Hz, 1H), 7.63 (d, *J* = 6.8 Hz, 1H), 7.72 (s, 1H), 7.81 (d, *J* = 8.8 Hz, 2H), 8.09 (d, *J* = 8.4 Hz, 1H), 10.62 (s, NH), 11.37 (s, NH). HRMS (ESI) calcd. for C_24_H_18_BrN_3_O_3_S: [M + Na]^+^ m/z: 530.0150, found: 530.0138.

### 5-bromo-N-(2-methyl-6-((4-(2-oxopyridin-1(2H)-yl)ph-enyl)carbamoyl)phenyl)thiophene-2-carboxamide (6a-c)

White solid product (0.25 g, 52%), m.p. > 250°C. ^1^H NMR (400 MHz, DMSO): δ ppm 2.26 (s, CH_3_), 6.28 (t, *J* = 6.4 Hz, 1H), 6.45 (d, *J* = 9.2 Hz, 1H), 7.30–7.38 (m, 4H), 7.46–7.75 (m, 3H), 7.59 (d, *J* = 6.8 Hz, 1H), 7.75–7.83 (m, 3H), 10.08 (s, NH), 10.41 (s, NH). HRMS (ESI) calcd. for C_24_H_18_BrN_3_O_3_S: [M + Na]^+^ m/z: 530.0150, found: 530.0138.

### 5-bromo-N-(2-((4-(3-oxomorpholino)phenyl)carbamo-yl)phenyl)thiophene-2-carboxamide (6a-d)

White solid product (0.30 g, 63%), m.p. > 250°C. ^1^H NMR (400 MHz, DMSO): δ ppm 3.72 (t, *J* = 4.8 Hz, CH_2_), 3.97 (t, *J* = 4.8 Hz, CH_2_), 4.19 (s, CH_2_), 7.30 (t, *J* = 7.6 Hz, 1H), 7.38 (d, *J* = 8.0 Hz, 3H), 7.56–7.62 (m, 2H), 7.72 (d, *J* = 8.8 Hz, 2H), 7.91 (d, *J* = 7.6 Hz, 1H), 8.25 (d, *J* = 8.4 Hz, 1H), 10.57 (s, NH), 11.60 (s, NH). HRMS (ESI) calcd. for C_22_H_18_BrN_3_O_4_S: [M + Na]^+^ m/z: 522.0099, found: 522.0098.

### 5-bromo-N-(4-chloro-2-((4-(3-oxomorpholino)phenyl) carbamoyl)phenyl)thiophene-2-carboxamide (6a-e)

Yellow solid product (0.29 g, 57%), m.p. > 250°C. ^1^H NMR (400 MHz, DMSO): δ ppm 3.72 (t, *J* = 4.8 Hz, CH_2_), 3.97 (t, *J* = 4.8 Hz, CH_2_), 4.19 (s, CH_2_), 7.37–7.40 (m, 3H), 7.58 (d, *J* = 4.0 Hz, 1H), 7.65–7.71 (m, 3H), 7.95 (s, 1H), 8.22 (d, *J* = 9.2 Hz, 1H), 10.63 (s, NH), 11.48 (s, NH). HRMS (ESI) calcd. for C_22_H_17_BrClN_3_O_4_S: [M + Na]^+^ m/z: 555.9709, found: 555.9707.

### 5-bromo-N-(4-methyl-2-((4-(3-oxomorpholino)phenyl) carbamoyl)phenyl)thiophene-2-carboxamide (6a-f)

White solid product (0.24 g, 49%), m.p. > 250°C. ^1^H NMR (400 MHz, DMSO): δ ppm 2.60 (s, CH_3_), 3.72 (t, *J* = 4.8 Hz, CH_2_), 3.97 (t, *J* = 4.8 Hz, CH_2_), 4.19 (s, CH_2_), 7.47–7.53 (m, 4H), 7.67 (d, *J* = 4.0 Hz, 1H), 7.82 (d, *J* = 8.4 Hz, 3H), 8.23 (d, *J* = 8.4 Hz, 1H), 10.64 (s, NH), 11.56 (s, NH). HRMS (ESI) calcd. for C_23_H_20_BrN_3_O_4_S: [M + Na]^+^ m/z: 536.0256, found: 536.0256.

### 5-bromo-N-(2-methyl-6-((4-(3-oxomorpholino)phenyl) carbamoyl)phenyl)thiophene-2-carboxamide (6a-g)

White solid product (0.27 g, 56%), m.p. > 250°C. ^1^H NMR (400 MHz, DMSO): δ ppm 2.25 (s, CH_3_), 3.68 (t, *J* = 4.8 Hz, CH_2_), 3.95 (t, *J* = 4.8 Hz, CH_2_), 4.17 (s, CH_2_), 7.28–7.34 (m, 4H), 7.44–7.48 (m, 2H), 7.66 (d, *J* = 8.4 Hz, 2H), 7.70 (d, *J* = 4.0 Hz, 1 H), 10.06 (s, NH), 10.28 (s, NH). HRMS (ESI) calcd. for C_23_H_20_BrN_3_O_4_S: [M + Na]^+^ m/z: 536.0256, found: 536.0242.

### 6-chloro-N-(4-chloro-2-((4-(2-oxopyridin-1(*2H*)-yl)ph-enyl)carbamoyl)phenyl)nicotinamide (6h-a)

White solid product (0.33 g, 73%), m.p. > 250°C. ^1^H NMR (400 MHz, DMSO): δ ppm 6.30 (t, *J* = 6.8 Hz, 1H), 6.46 (d, *J* = 9.2 Hz, 1H), 7.37 (d, *J* = 8.8 Hz, 2H), 7.49 (t, *J* = 8.8 Hz, 1H), 7.62 (d, *J* = 8.4 Hz, 2H), 7.72 (d, *J* = 8.4 Hz, 2H), 7.81 (d, *J* = 6.8 Hz, 2H), 7.94 (s, 1H), 8.20 (d, *J* = 8.4 Hz, 1H), 8.27 (d, *J* = 8.0 Hz, 1H), 8.89 (s, 1H), 10.73 (s, NH), 11.37 (s, NH). HRMS (ESI) calcd. for C_24_H_16_Cl_2_N_4_O_3_: [M + Na]^+^ m/z: 501.0497, found: 501.0494.

### 6-chloro-N-(4-methyl-2-((4-(2-oxopyridin-1(*2H*)-yl)ph-enyl)carbamoyl)phenyl)nicotinamide (6h-b)

White solid product (0.29 g, 67%), m.p. > 250°C. ^1^H NMR (400 MHz, DMSO): δ ppm 2.40 (s, CH_3_), 6.30 (t, *J* = 6.8 Hz, 1H), 6.46 (d, *J* = 9.2 Hz, 1H), 7.37 (d, *J* = 8.8 Hz, 2H), 7.43 (d, *J* = 8.0 Hz, 1H), 7.49 (t, *J* = 8.8 Hz, 1H), 7.62 (d, *J* = 6.8 Hz, 1H), 7.72 (d, *J* = 8.4 Hz, 2H), 7.80 (d, *J* = 8.8 Hz, 2H), 8.08 (d, *J* = 8.4 Hz, 1H), 8.26 (d, *J* = 8.4 Hz, 1H), 8.88 (s, 1H), 10.62 (s, NH), 11.34 (s, NH). HRMS (ESI) calcd. for C_25_H_19_ClN_4_O_3_: [M + Na]^+^ m/z: 481.1043, found: 481.1037.

### 6-chloro-N-(2-methyl-6-((4-(2-oxopyridin-1(2H)-yl)ph-enyl)carbamoyl)phenyl)nicotinamide (6h-c)

White solid product (0.27 g, 62%), m.p. > 250°C. ^1^H NMR (400 MHz, DMSO): δ ppm 2.28 (s, CH_3_), 6.28 (t, *J* = 6.8 Hz, 1H), 6.44 (d, *J* = 8.8 Hz, 1H), 7.30 (d, *J* = 8.8 Hz, 2H), 7.34 (d, *J* = 8.8 Hz, 1H), 7.45–7.51 (m, 3H), 7.58 (d, *J* = 8.8 Hz, 1H), 7.67 (d, *J* = 8.4 Hz, 1H), 7.76 (d, *J* = 8.4 Hz, 2H), 8.29 (d, *J* = 8.0 Hz, 1H), 8.89 (s, 1H), 10.23 (s, NH), 10.47 (s, NH). HRMS (ESI) calcd. for C_25_H_19_ClN_4_O_3_: [M + Na]^+^ m/z: 481.1043, found: 481.1039.

### 6-chloro-N-(2-((4-(3-oxomorpholino)phenyl)carbamo-yl)phenyl)nicotinamide (6h-d)

White solid product (0.30 g, 69%), m.p. > 250°C. ^1^H NMR (400 MHz, DMSO): δ ppm 3.71 (t, *J* = 4.8 Hz, CH_2_), 3.97 (t, *J* = 4.8 Hz, CH_2_), 4.19 (s, CH_2_), 7.36 (d, *J* = 8.8 Hz, 3H), 7.62 (t, *J* = 7.2 Hz, 1H), 7.70–7.73 (m, 3H), 7.89 (d, *J* = 7.6 Hz, 1H), 8.24–8.29 (m, 2H), 8.89 (m, 1H), 10.56 (s, NH), 11.54 (s, NH). HRMS (ESI) calcd. for C_23_H_19_ClN_4_O_4_: [M + Na]^+^ m/z: 473.0993, found: 473.0987.

### 6-chloro-N-(4-chloro-2-((4-(3-oxomorpholino)phenyl) carbamoyl)phenyl)nicotinamide (6h-e)

Yellow solid product (0.28 g, 61%), m.p. > 250°C. ^1^H NMR (400 MHz, DMSO): δ ppm 3.71 (t, *J* = 4.8 Hz, CH_2_), 3.97 (t, *J* = 4.8 Hz, CH_2_), 4.19 (s, CH_2_), 7.37 (d, *J* = 8.8 Hz, 2H), 7.67–7.74 (m, 4H), 7.93 (s, 1H), 8.20–8.27 (m, 2H), 8.88 (m, 1H), 10.63 (s, NH), 11.43 (s, NH). HRMS (ESI) calcd. for C_23_H_18_Cl_2_N_4_O_4_: [M + Na]^+^ m/z: 507.0603, found: 507.0594.

### 6-chloro-N-(4-methyl-2-((4-(3-oxomorpholino)phenyl) carbamoyl)phenyl)nicotinamide (6h-f)

White solid product (0.28 g, 64%), m.p. > 250°C. ^1^H NMR (400 MHz, DMSO): δ ppm 2.39 (s, CH_3_), 3.71 (t, *J* = 4.8 Hz, CH_2_), 3.97 (t, *J* = 4.8 Hz, CH_2_), 4.19 (s, CH_2_), 7.36 (d, *J* = 8.4 Hz, 2H), 7.43 (d, *J* = 8.8 Hz, 1H), 7.71 (d, *J* = 8.8 Hz, 4H), 8.11 (d, *J* = 8.4 Hz, 1H), 8.25 (d, *J* = 8.0 Hz, 1H), 8.88 (s, 1H), 10.52 (s, NH), 11.41 (s, NH). HRMS (ESI) calcd. for C_24_H_21_ClN_4_O_4_: [M + Na]^+^ m/z: 487.1149, found: 487.1148.

### 6-chloro-N-(2-methyl-6-((4-(3-oxomorpholino)phenyl) carbamoyl)phenyl)nicotinamide (6h-g)

White solid product (0.27 g, 62%), m.p. > 250°C. ^1^H NMR (400 MHz, DMSO): δ ppm 2.27 (s, CH_3_), 3.71 (t, *J* = 4.8 Hz, CH_2_), 3.97 (t, *J* = 4.8 Hz, CH_2_), 4.17 (s, CH_2_), 7.27–7.34 (m, 3H), 7.45–7.51 (m, 2H), 7.65–7.73 (m, 3H), 8.28 (d, *J* = 8.0 Hz, 1H), 8.89 (s, 1H), 10.10 (s, NH), 10.21 (s, NH). HRMS (ESI) calcd. for C_24_H_21_ClN_4_O_4_: [M + Na]^+^ m/z: 487.1149, found: 487.1146.

### 2,4-dichloro-N-(4-chloro-2-((4-(2-oxopyridin-1(*2H*)-yl) phenyl)carbamoyl)phenyl)benzamide (6k-a)

White solid product (0.27 g, 59%), m.p. > 250°C. ^1^H NMR (400 MHz, DMSO): δ ppm 6.29 (t, *J* = 6.8 Hz, 1H), 6.46 (d, *J* = 9.2 Hz, 1H), 7.36 (d, *J* = 8.0 Hz, 2H), 7.49 (t, *J* = 8.8 Hz, 1H), 7.56–7.61 (m, 2H), 7.64–7.68 (m, 2H), 7.74–7.79 (m, 2H), 7.87 (s, 1H), 8.10 (d, *J* = 7.6 Hz, 1H), 10.69 (s, NH), 10.94 (s, NH). HRMS (ESI) calcd. for C_25_H_16_Cl_3_N_3_O_3_: [M + Na]^+^ m/z: 534.0155, found: 534.0146.

### 2,4-dichloro-N-(4-methyl-2-((4-(2-oxopyridin-1(*2H*)-yl) phenyl)carbamoyl)phenyl)benzamide (6k-b)

Yellow solid product (0.27 g, 60%), m.p. > 250°C. ^1^H NMR (400 MHz, DMSO): δ ppm 2.39 (s, CH_3_), 6.29 (t, *J* = 6.8 Hz, 1H), 6.46 (d, *J* = 8.8 Hz, 1H), 7.35 (d, *J* = 8.8 Hz, 2H), 7.42 (d, *J* = 8.4 Hz, 1H), 7.49 (t, *J* = 8.8 Hz, 1H), 7.55 (d, *J* = 8.0 Hz, 1H), 7.59–7.65 (m, 3H), 7.73 (s, 1H), 7.79 (d, *J* = 8.8 Hz, 2H), 8.00 (d, *J* = 8.4 Hz, 1H), 10.58 (s, NH), 10.85 (s, NH). HRMS (ESI) calcd. for C_26_H_19_Cl_2_N_3_O_3_: [M + Na]^+^ m/z: 514.0701, found: 514.0697.

### 2-(2,4-dichlorobenzamido)-3-methyl-N-(4-(2-oxopyrid-in-1(2H)-yl)phenyl)benzamide (6k-c)

White solid product (0.26 g, 58%), m.p. > 250°C. ^1^H NMR (400 MHz, DMSO): δ ppm 2.34 (s, CH_3_), 6.30 (t, *J* = 6.8 Hz, 1H), 6.46 (d, *J* = 9.2 Hz, 1H), 7.34 (d, *J* = 8.8 Hz, 3H), 7.49–7.57 (m, 5H), 7.61 (d, *J* = 8.4 Hz, 1H), 7.69 (s, 1H), 7.85 (d, *J* = 8.4 Hz, 2H), 10.11 (s, NH), 10.48 (s, NH). HRMS (ESI) calcd. for C_26_H_19_Cl_2_N_3_O_3_: [M + Na]^+^ m/z: 514.0701, found: 514.0699.

### 2,4-dichloro-N-(2-((4-(3-oxomorpholino)phenyl)carba-moyl)phenyl)benzamide (6k-d)

White solid product (0.31 g, 71%), m.p. > 250°C. ^1^H NMR (400 MHz, DMSO): δ ppm 3.69 (t, *J* = 4.8 Hz, CH_2_), 3.96 (t, *J* = 4.8 Hz, CH_2_), 4.18 (s, CH_2_), 7.33 (d, *J* = 8.4 Hz, 3H), 7.55–7.61 (m, 2H), 7.65–7.74 (m, 4H), 7.82 (d, *J* = 8.0 Hz, 1H), 8.17 (d, *J* = 7.2 Hz, 1H), 10.51 (s, NH), 11.02 (s, NH). HRMS (ESI) calcd. for C_24_H_19_Cl_2_N_3_O_4_: [M + Na]^+^ m/z: 506.0650, found: 506.0646.

### 2,4-dichloro-N-(4-chloro-2-((4-(3-oxomorpholino)phe-nyl)carbamoyl)phenyl)benzamide (6k-e)

Yellow solid product (0.29 g, 62%), m.p. > 250°C. ^1^H NMR (400 MHz, DMSO): δ ppm 3.70 (t, *J* = 4.8 Hz, CH_2_), 3.96 (t, *J* = 4.8 Hz, CH_2_), 4.18 (s, CH_2_), 7.35 (d, *J* = 8.8 Hz, 2H), 7.57 (d, *J* = 8.4 Hz, 1H), 7.64–7.70 (m, 4H), 7.74 (s, 1H), 7.86 (s, 1H), 8.13 (d, *J* = 8.8 Hz, 1H), 10.59 (s, NH), 10.97 (s, NH). HRMS (ESI) calcd. for C_24_H_18_Cl_3_N_3_O_4_: [M + Na]^+^ m/z: 540.0261, found: 540.0269.

### 2,4-dichloro-N-(4-methyl-2-((4-(3-oxomorpholino)phe-nyl)carbamoyl)phenyl)benzamide (6k-f)

Yellow solid product (0.31 g, 70%), m.p. > 250°C. ^1^H NMR (400 MHz, DMSO): δ ppm 3.69 (t, *J* = 4.8 Hz, CH_2_), 3.96 (t, *J* = 4.8 Hz, CH_2_), 4.18 (s, CH_2_), 7.34 (d, *J* = 8.8 Hz, 2H), 7.41 (d, *J* = 8.0 Hz, 1H), 7.55 (d, J = 8.4 Hz, 1H), 7.63–7.73 (m, 4H), 8.05 (d, *J* = 8.4 Hz, 1H), 10.47 (s, NH), 10.89 (s, NH). HRMS (ESI) calcd. for C_25_H_21_Cl_2_N_3_O_4_: [M + Na]^+^ m/z: 520.0807, found: 520.0802.

### 2-(2,4-dichlorobenzamido)-3-methyl-N-(4-(3-oxomor-pholino)phenyl)benzamide (6k-g)

White solid product (0.31 g, 69%), m.p. > 250°C. ^1^H NMR (400 MHz, DMSO): δ ppm 3.70 (t, *J* = 4.8 Hz, CH_2_), 3.96 (t, *J* = 4.8 Hz, CH_2_), 4.18 (s, CH_2_), 7.31–7.35 (m, 3H), 7.44 (d, *J* = 7.6 Hz, 2H), 7.50–7.56 (m, 2H), 7.68 (s, 1H), 7.75 (d, *J* = 8.8 Hz, 2H), 10.08 (s, NH), 10.35 (s, NH). HRMS (ESI) calcd. for C_25_H_21_Cl_2_N_3_O_4_: [M + Na]^+^ m/z: 520.0807, found: 520.0800.

### 5-chloro-2-(4-methoxybenzamido)-N-(4-(2-oxopyridin-1(*2H*)-yl)phenyl)benzamide (6l-a)

White solid product (0.24 g, 57%), m.p. > 250°C. ^1^H NMR (400 MHz, DMSO): δ ppm 3.82 (s, CH_3_), 6.31 (t, *J* = 6.4 Hz, 1H), 6.47 (d, *J* = 9.6 Hz, 1H), 7.10 (d, *J* = 8.8 Hz, 2H), 7.40 (d, *J* = 8.8 Hz, 2H), 7.50 (t, *J* = 8.8 Hz, 1H), 7.62–7.68 (m, 2H), 7.80 (d, *J* = 8.4 Hz, 2H), 7.88 (d, *J* = 8.8 Hz, 2H), 7.99 (s, 1H), 8.46 (d, *J* = 9.2 Hz, 1H), 10.75 (s, NH), 11.49 (s, NH). HRMS (ESI) calcd. for C_26_H_20_ClN_3_O_4_: [M + Na]^+^ m/z: 496.1040, found: 496.1037.

### 2-(4-methoxybenzamido)-5-methyl-N-(4-(2-oxopyrid-in-1(*2H*)-yl)phenyl)benzamide (6l-b)

White solid product (0.29 g, 71%), m.p. > 250°C. ^1^H NMR (400 MHz, DMSO): δ ppm 2.39 (s, CH_3_), 3.82 (s, CH_3_), 6.31 (t, *J* = 6.4 Hz, 1H), 6.47 (d, *J* = 9.2 Hz, 1H), 7.10 (d, *J* = 8.8 Hz, 2H), 7.38–7.44 (m, 3H), 7.50 (t, *J* = 7.2 Hz, 1H), 7.63 (d, *J* = 6.0 Hz, 1H), 7.75 (s, 1H), 7.81 (d, *J* = 8.4 Hz, 2H), 7.87 (d, *J* = 8.4 Hz, 2H), 8.35 (d, *J* = 8.4 Hz, 2H), 10.65 (s, NH), 11.44 (s, NH). HRMS (ESI) calcd. for C_27_H_23_N_3_O_4_: [M + Na]^+^ m/z: 476.1586, found: 476.1582.

### 2-(4-methoxybenzamido)-3-methyl-N-(4-(2-oxopyrid-in-1(2H)-yl)phenyl)benzamide (6l-c)

White solid product (0.30 g, 73%), m.p. > 250°C. ^1^H NMR (400 MHz, DMSO): δ ppm 2.26 (s, CH_3_), 3.81 (s, CH_3_), 6.27 (t, *J* = 6.8 Hz, 1H), 6.44 (d, *J* = 9.2 Hz, 1H), 7.01 (d, *J* = 8.8 Hz, 2H), 7.28–7.36 (m, 3H), 7.45–7.49 (m, 3H), 7.57 (d, *J* = 6.4 Hz, 1H), 7.76 (d, *J* = 8.8 Hz, 2H), 7.92 (d, *J* = 8.4 Hz, 2H), 9.85 (s, NH), 10.38 (s, NH). HRMS (ESI) calcd. for C_27_H_23_N_3_O_4_: [M + Na]^+^ m/z: 476.1586, found: 476.1585.

### 2-(4-methoxybenzamido)-N-(4-(3-oxomorpholino)phe-nyl)benzamide (6l-d)

White solid product (0.29 g, 72%), m.p. > 250°C. ^1^H NMR (400 MHz, DMSO): δ ppm 3.72 (t, *J* = 4.8 Hz, CH_2_), 3.83 (s, CH_3_), 3.97 (t, *J* = 4.8 Hz, CH_2_), 4.19 (s, CH_2_), 7.10 (d, *J* = 8.4 Hz, 2H), 7.26 (t, *J* = 7.6 Hz, 1H), 7.38 (d, *J* = 8.8 Hz, 2H), 7.60 (t, *J* = 8.0 Hz, 1H), 7.72 (d, *J* = 8.8 Hz, 2H), 7.87–7.93 (m, 3H), 8.50 (d, *J* = 8.0 Hz, 1H), 10.58 (s, NH), 11.64 (s, NH). HRMS (ESI) calcd. for C_25_H_23_N_3_O_5_: [M + Na]^+^ m/z: 468.1535, found: 468.1535.

### 5-chloro-2-(4-methoxybenzamido)-N-(4-(3-oxomorph-olino)phenyl)benzamide (6l-e)

Yellow solid product (0.23 g, 54%), m.p. > 250°C. ^1^H NMR (400 MHz, DMSO): δ ppm 3.72 (t, *J* = 4.8 Hz, CH_2_), 3.82 (s, CH_3_), 3.97 (t, *J* = 4.8 Hz, CH_2_), 4.19 (s, CH_2_), 7.10 (d, *J* = 8.8 Hz, 2H), 7.39 (d, *J* = 8.8 Hz, 2H), 7.65–7.62 (m, 3H), 7.87 (d, *J* = 8.8 Hz, 2H), 7.97 (s, 1H), 7.48 (d, *J* = 8.8 Hz, 1H), 10.66 (s, NH), 11.53 (s, NH). HRMS (ESI) calcd. for C_25_H_22_ClN_3_O_5_: [M + Na]^+^ m/z: 502.1146, found: 502.1144.

### 2-(4-methoxybenzamido)-5-methyl-N-(4-(3-oxomorph-olino)phenyl)benzamide (6l-f)

White solid product (0.28 g, 68%), m.p. > 250°C. ^1^H NMR (400 MHz, DMSO): δ ppm 3.72 (t, *J* = 4.8 Hz, CH_2_), 3.82 (s, CH_3_), 3.97 (t, *J* = 4.8 Hz, CH_2_), 4.19 (s, CH_2_), 7.09 (d, *J* = 8.8 Hz, 2H), 7.37–7.43 (m, 3H), 7.70–7.73 (m, 3H), 7.86 (d, *J* = 8.8 Hz, 2H), 8.37 (d, *J* = 8.4 Hz, 1H), 10.54 (s, NH), 11.49 (s, NH). HRMS (ESI) calcd. for C_26_H_25_N_3_O_5_: [M + Na]^+^ m/z: 482.1692, found: 482.1686.

### 2-(4-methoxybenzamido)-3-methyl-N-(4-(3-oxomorph-olino)phenyl)benzamide (6l-g)

White solid product (0.29 g, 70%), m.p. > 250°C. ^1^H NMR (400 MHz, DMSO): δ ppm 3.66 (t, *J* = 4.8 Hz, CH_2_), 3.80 (s, CH_3_), 3.94 (t, *J* = 4.8 Hz, CH_2_), 4.16 (s, CH_2_), 7.01 (d, *J* = 8.8 Hz, 2H), 7.27–7.32 (m, 3H), 7.44 (d, *J* = 8.4 Hz, 2H), 7.66 (d, *J* = 8.8 Hz, 2H), 7.91 (d, *J* = 8.4 Hz, 2H), 9.85 (s, NH), 10.26 (s, NH). HRMS (ESI) calcd. for C_26_H_25_N_3_O_5_: [M + Na]^+^ m/z: 482.1692, found: 482.1683.

### Inhibition activity measurement against FXa

The inhibition of FXa was measured using human FXa (Hyphen BioMed, city, Paris, FRA) and chromogenic substrate CS-11(22) (Hyphen BioMed, Paris, FRA) in 384-well microtiter plates at room temperature. The synthesized compounds 6 and rivaroxaban were dissolved in DMSO at a concentration of 10 mM and then serially diluted to spanning a range of 30 nM to 100 μM, respectively. 2 μL of FXa (56.8 nM), 16 μL of Tris buffer (adjust to pH 7.4 with HCl containing 0.3 M NaCl and 50 mM Tris) and 3 μL of test compound were added to the well, respectively. The negative control was composed of the same mixed solutions except replacing test compound with DMSO. The positive control was composed of the same mixed solutions except replacing test compound with rivaroxaban. After incubated at 37°C for 5 min, 8 μL of FXa substrate solution (3.5 mM) was added and then incubated at 37°C for 25 min. The FXa activity was measured at 405 nm using a SpectraMax M5 (Molecular Devices, Sunnyvale, CA, USA). The IC_50_ was calculated by the software named SPSS (IBM, North Castle, NY, USA) and the Probit function in it.

### Thrombin inhibition activity of 6a, 6a-b, 6a-e, 6k, 6k-a and 6k-b

The inhibition of thrombin was measured using human FIIa (Hyphen BioMed, Paris, FRA) and chromogenic substrate CS-01(38) (Hyphen BioMed, Paris, FRA) in 384-well microtiter plates at room temperature. The compounds 6a, 6a-b, 6a-e, 6k, 6k-a, 6k-b and rivaroxaban were dissolved in DMSO to a concentration of 10 mM and then serially diluted to spanning a range of 10 μM to 100 μM, respectively. 2 μL of FIIa (3 NIH/mL), 20 μL of Tris buffer (adjust to pH 7.4 with HCl) containing 0.3 M NaCl and 50 mM Tris and 2 μL of test compound were added to the well, respectively. The negative control was composed of the same mixed solutions except replacing test compound with DMSO. The positive control was composed of the same mixed solutions except replacing test compound with rivaroxaban. After incubated at 37°C for 5 min, 3 μL of FIIa substrate solution (4 mM) was added and then incubated at 37°C for 25 min. The FIIa activity was measured at 405 nm using a SpectraMax M5 (Molecular Devices).

### Prothrombin time (PT) assay

A commercially available automatic coagulometer (Steellex Science Instrument Co., Ltd., Beijing, China) was employed to measure PT. The clotting times were also measured using the instrument itself, in accordance with the manufacturer's instructions. Increasing concentrations of inhibitor or solvent were added to human (39 Years old, male, Chinese) plasma and incubated for 3 min at 37°C. Prothrombin time (PT) was determined by automatic coagulometer.

### Docking simulation

FXa structure was selected from the protein data bank (PDB code: 2xbv) and prepared using Protein Preparation Wizard in Schrödinger package, including assigning bond orders, adding hydrogen atoms, deleting water molecules, creating disulfide bonds and capping terminals. The original ligand of the protein structure-XBV was used as the docking center to generate the receptor grid parameters. The box size was set as 12 Å. Compounds 6a, 6a-b, 6a-e, 6k, 6k-a and 6k-b were prepared using the LigPrep module in Schrödinger. Epik method was used to determine possible ionization state of ligands at pH 7.0 ± 2.0 and low-energy conformers were produced using OPLS-2005 force field. Molecular docking calculations were performed by using Glide module with default parameters at standard precision in Schrödinger.

## CONCLUSIONS

In conclusion, the synthetic anthranilamide compounds were evaluated as novel inhibitors of FXa. Among 43 compounds, 6a, 6a-b, 6a-e, 6k, 6k-a and 6k-b showed excellent activity and selectivity over thrombin. In further, the compound 6a-e showed best anticoagulant activity (3.8 μM) in the six compounds significantly. The computational docking simulation study clarified the interactions mode of compounds. The results in this study indicated that compound 6a-e exhibited remarkable thrombin inhibitory effect in intro and it might be a potent novel anti-coagulator for further *in vivo* studies.
